# Infantile ruptured acom aneurysm treated with endovascular stent-coil embolization: case report

**DOI:** 10.1186/s12883-025-04366-3

**Published:** 2025-08-13

**Authors:** Yigit Can Senol, Halis Emre Ciftci, Naime Dilara Ozkan, Bige Sayin, Ergun Daglioglu

**Affiliations:** 1 Department of Neurologic Surgery, University of California, San Francisco, 513 Parnassus Ave, San Francisco, 94143 USA; 2 Department of Neurological Surgery, University of Health Sciences, Ankara Bilkent City Hospital, Ankara, Turkey; 3 Department of Radiology, University of Health Sciences, Ankara Bilkent City Hospital, Ankara, Turkey

**Keywords:** Anterior communicating aneurysm, Infant, Stent-coil, Pediatric

## Abstract

A 15-month-old infant presented with an episode of acute agitation, characterized by crying, refusal to feed, and a focal seizure involving the left arm and leg lasting 1–2 min. Following the seizure, the infant fell asleep but experienced another brief seizure during transport to the hospital. Initial assessment at a private hospital in Mersin, Turkey, led to a referral to our center, where imaging revealed an intraparenchymal hemorrhage in the left frontobasal region. The hemorrhage extended into all ventricles and the right retrosellar area, with notable rightward shift and ventricular enlargement. CTA confirmed a ruptured Acom aneurysm with a bleb. Diagnostic angiography was performed, and an endovascular stent coiling procedure was performed without hematoma evacuation. At 1-year follow-up, patients' symptoms improved without neurological sequelae, and MR angiography revealed no residual filling in the aneurysm.

## Taking home message

- Endovascular treatment is a viable alternative to surgical clipping for pediatric ruptured cerebral aneurysms, offering reduced surgical trauma and faster recovery, though long-term durability remains an area of ongoing research.
- Stent-assisted coiling provides effective aneurysm occlusion while preserving the parent artery, but requires careful consideration of antiplatelet therapy, especially in cases with concurrent hemorrhage.
- Long-term follow-up is essential to monitor for aneurysm recurrence, in-stent stenosis, and vascular remodeling, ensuring optimal outcomes in the pediatric population.

## Introduction

Pediatric cerebral aneurysms are rare (1.6–7% of all intracerebral aneurysms), and similar to the adult population, ruptured aneurysms can be life-threatening in children [1, 2]. The etiology of cerebral aneurysms in the pediatric population includes congenital malformations, genetic predisposition, infections, trauma, and hormonal influences. Identifying the underlying cause is important, as it may influence both treatment decisions and long-term management strategies [3, 4]. Diagnosis and treatment of pediatric cerebral aneurysms are essential because ruptured pediatric cerebral aneurysms that are left open or incompletely treated have higher rates of death due to rebleeding in the first 10 years after initial hemorrhage [3]. This case underscores the endovascular embolization and stent-coil procedures could be a viable alternative treatment for ruptured intracerebral aneurysms.

### Case presentation

15-month-old infant presented with a focal seizure affecting the right arm and leg, lasting 1–2 min. Following the seizure, the patient became drowsy but experienced another brief seizure during transport to the hospital. Initial evaluation at a private hospital in the outer center, led to a referral to our center, where imaging revealed an intraparenchymal hemorrhage in the left Frontobasal region. The patient was drowsy, with eyes opening in response to verbal stimuli and spontaneous extremity movements, with reduced movements on the right side present. The patient was transferred to the pediatric intensive care unit for further diagnostic evaluation, including advanced testing and CT angiography.

**Fig. 1:**
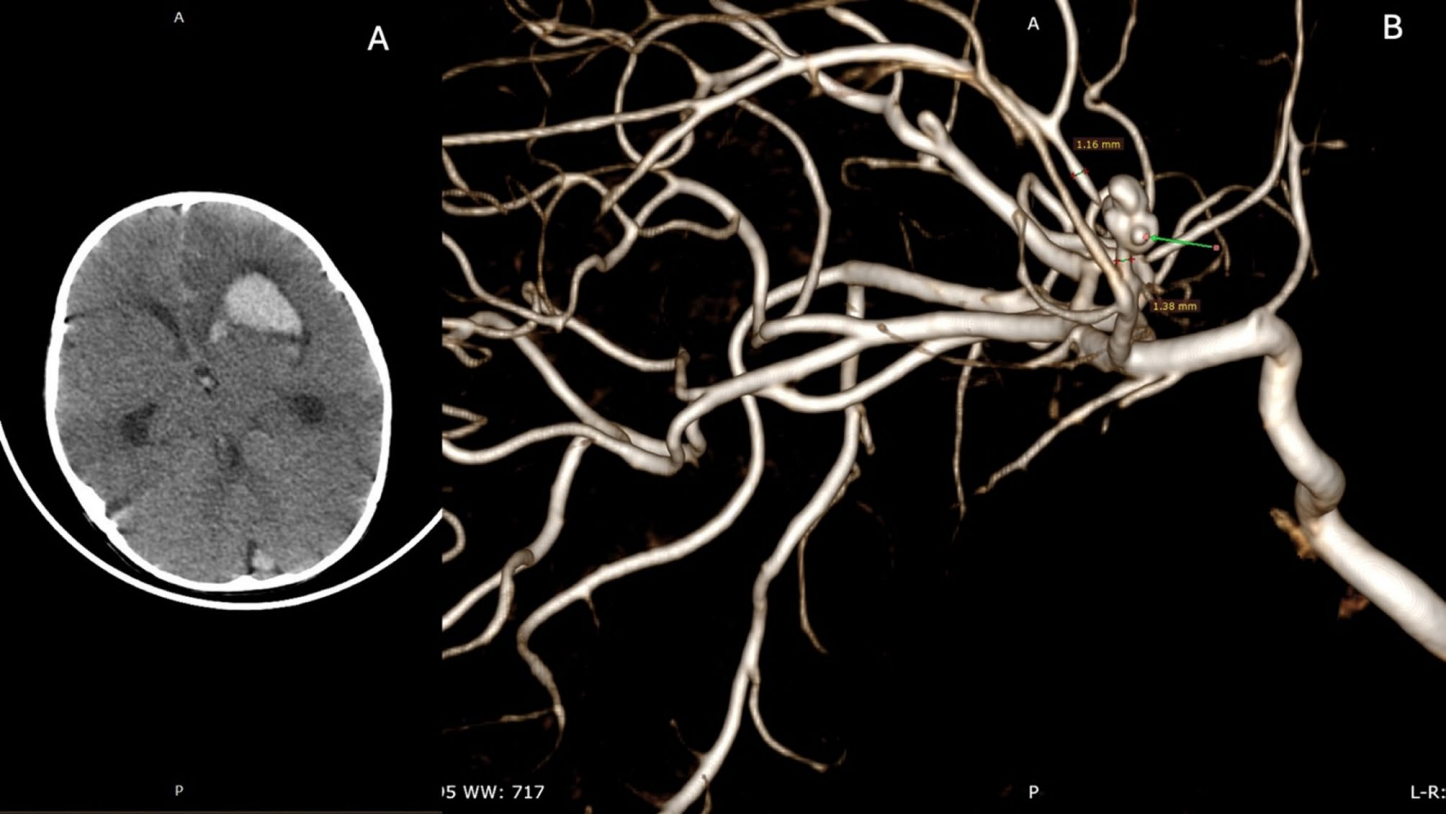
A: Preoperative CT demonstrates left frontobasal hematoma extended to ventricles B: After CT angiography revealed that an Acom aneurysm with multiple blebs is the cause of the parenchymal hemorrhage

CT angiography revealed that the hemorrhage extended into all ventricles and the right retrosellar area, with a notable rightward shift and ventricular enlargement (Fig. 1A). CTA confirmed a ruptured anterior communicating (ACom) artery aneurysm with a bleb (Fig. 1B). A ruptured ACom aneurysm was confirmed as the cause of the intraparenchymal hemorrhage. However, since CT angiography is not the gold standard for diagnosing cerebrovascular malformations, a vascular anomaly potentially contributing to the hemorrhage could have been missed. Spontaneous intraparenchymal hemorrhage is relatively rare in this age group; therefore, hemorrhagic stroke or arteriovenous malformations were ruled out after the initial diagnosis was confirmed with CT angiography.

**Fig. 2:**
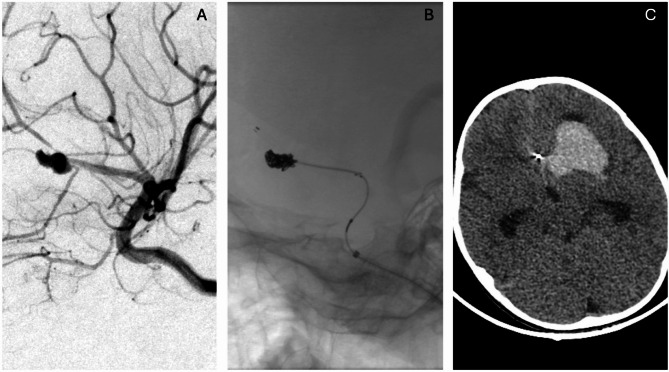
A: A roadmap image shows the preoperative subtracted image, A1 artery size is decreased possibly vasospasm after subarachnoid hemorrhage B: A single stent-assisted coiling is completed in native images. C: Postoperative CT demonstrates no increase in hemorrhage volume and no signs of acute hydrocephalus

The primary treatment options for this case included surgical aneurysm clipping with hematoma evacuation and an endovascular approach. The procedural risks associated with both strategies were thoroughly explained to the patient’s parents, allowing them to make an informed decision regarding the intervention. Following a comprehensive discussion with the family, an endovascular approach was decided, with an initial plan for primary coiling of the aneurysm. Due to a coil migration during the procedure, stent assistance was used (Fig. 2A). A single stent was deployed in the unilateral A1 branch, and microcatheter was navigated into the aneurysm for additional coil deployment (Fig. 2B).

Postoperatively, dual antiplatelet therapy (DAPT) was initiated using pediatric-specific dosing, consisting of clopidogrel 37.5 mg daily and aspirin 100 mg daily, based on regimens reported in the literature for children under 45 kg [5]. Clopidogrel was continued for six months and then discontinued, while aspirin was maintained as monotherapy. Due to the patient’s age, platelet function was monitored clinically and radiographically rather than with laboratory assays.

**Fig. 3:**
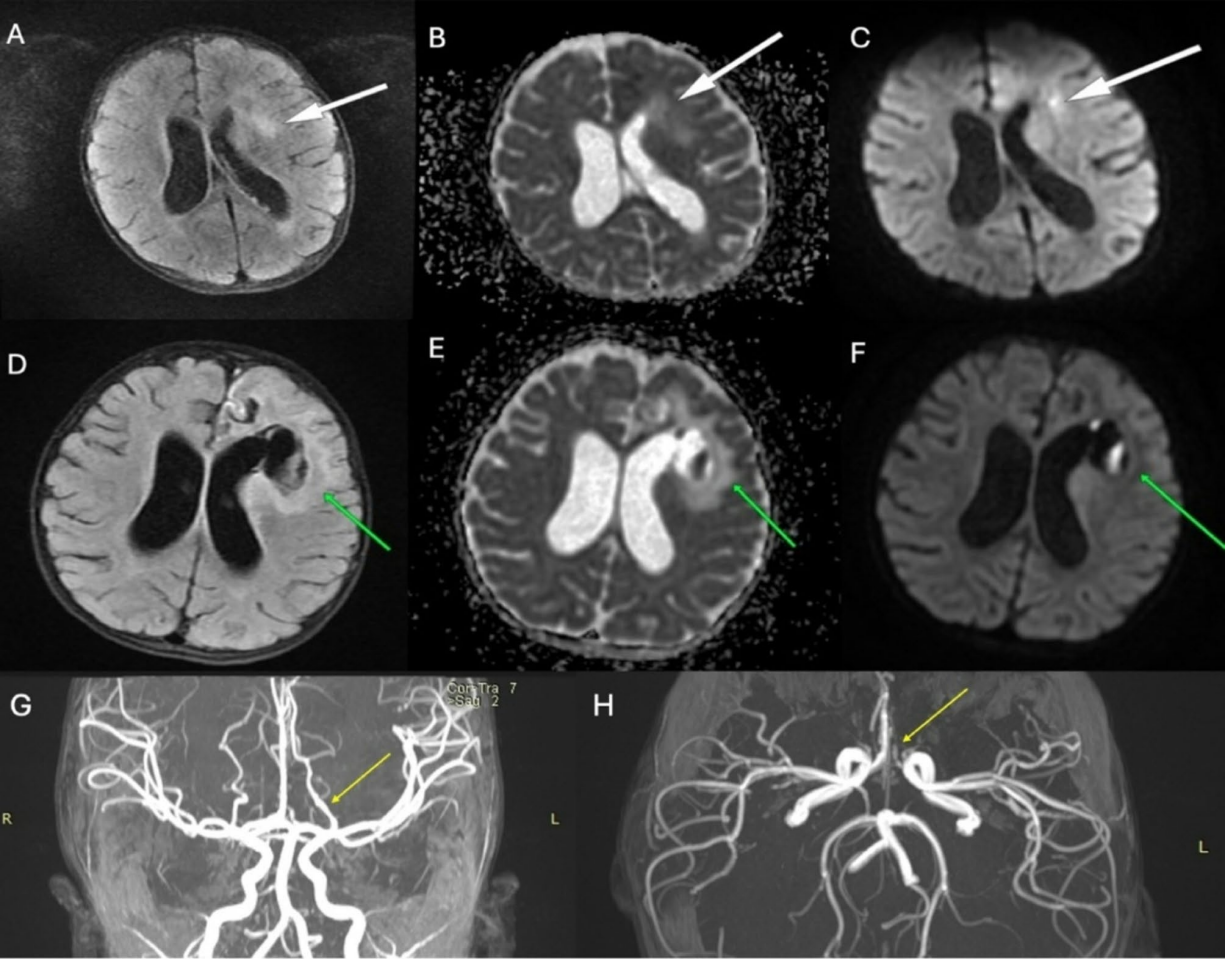
(A-B-C) Follow-up MRI showing hematoma resorption(White arrow) and no residual aneurysm filling (From left to right; T2, ADC, DWI). (D-E-F) Cerebral edema(Green arrow) near the hematoma was observed on the one-month follow-up MRI, though the patient’s neurological condition remained unchanged. (From left to right: T2, ADC, DWI). (G-H) MR angiography at 1-year follow-up revealed no residual aneurysm filling(yellow arrow)

Postoperative CT imaging showed no significant change in the hemorrhage (Fig. 2C), leading to the decision to defer hematoma evacuation. Sedation was gradually reduced, and the patient was successfully extubated. A 24-hour postoperative follow-up diffusion MRI indicated progressive hematoma resorption without evidence of new bleeding (Fig. 3A-B-C). The patient gradually transitioned to oral feeding and began physical therapy. Recovery was closely monitored with routine neurological assessments, and a structured rehabilitation program was implemented. A month follow-up MRI revealed hematoma resorption with no evidence of residual aneurysm filling (Fig. 3D-E-F). After a year later, follow-up MR angiography revealed no residual filling of an aneurysm(Fig. 3G-H).

## Discussion

This illustrative case report presents a ruptured anterior communicating (ACom) artery aneurysm in an infant successfully treated with single stent-assisted coiling. This case underscores that endovascular embolization and stent-coil procedures could be a viable alternative treatment for ruptured intracerebral aneurysms.

Endovascular treatment offers several advantages in the pediatric population, including reduced surgical trauma, avoidance of brain retraction, and shorter recovery times [1]. Stent-assisted coiling provides both immediate aneurysm occlusion and the potential for endothelialization over time, thereby reducing the risk of re-rupture as previously reported in posterior circulation aneurysms in pediatric population [6]. However, the requirement for long-term antiplatelet therapy remains a significant consideration, particularly in cases with concurrent hemorrhage. In this case, dual antiplatelet therapy with clopidogrel and ASA was initiated postoperatively with doses adjusted to the patient’s weight. A single stent was deployed in the unilateral A1 branch, allowing successful catheterization of the aneurysm for coil placement. Although surgical clipping remains a durable treatment option, its application in infants presents unique challenges. Craniotomy and direct aneurysm manipulation carry risks of brain retraction injury and vascular damage, particularly in small-caliber arteries. In contrast, endovascular techniques eliminate the need for extensive dissection and allow aneurysm occlusion without direct surgical exposure. However, concerns regarding the long-term patency of stents and their interaction with the developing vasculature in infants remain areas of ongoing research.

To our knowledge, this is one of the youngest reported cases of ruptured anterior communicating artery aneurysm treated successfully with stent-assisted coiling. In contrast to most prior pediatric reports that emphasize surgical clipping or primary coiling [7, 8], our case highlights the feasibility of stent use even in infants when anatomical and procedural conditions allow. However, it is important to note that stent placement is generally not recommended in children under the age of four, as the internal carotid and anterior cerebral arteries have not yet reached their final caliber, which may impact long-term device compatibility. Additionally, despite the presence of a substantial intraparenchymal hematoma, the patient demonstrated steady clinical improvement and progressive hematoma resorption without requiring surgical evacuation. This supports the notion that, with careful monitoring, conservative management may be appropriate in select cases. Furthermore, given the relatively higher recurrence rates associated with endovascular coiling—especially in pediatric patients—long-term angiographic follow-up is essential to monitor for potential recanalization or in-stent stenosis. Our findings contribute to the evolving literature on pediatric endovascular interventions and support the cautious but expanding use of advanced devices in very young patients.

## Conclusion

Our case highlights the evolving role of endovascular treatment in pediatric intracranial aneurysms. While surgical clipping remains a viable option, stent-assisted coiling provides a minimally invasive alternative that can achieve effective aneurysm occlusion with lower perioperative morbidity. Further studies and long-term follow-up are needed to establish standardized treatment protocols and assess the durability of endovascular interventions in the pediatric population.

## Data Availability

All data analyzed during this study are included in this manuscript and written informed consent was granted from patient’s legal guardians.
